# Fastening in Rock Mass—Structural Design of Shallow Embedded Anchors in Inhomogeneous Substrate

**DOI:** 10.3390/ma17246044

**Published:** 2024-12-10

**Authors:** Stefan Lamplmair-Irsigler, Oliver Zeman, Elisabeth Stierschneider, Klaus Voit

**Affiliations:** 1 Department of Civil Engineering and Natural Hazards, Institute of Applied Geology, University of Natural Resources and Life Sciences Vienna, Peter-Jordan-Street 82, 1190 Vienna, Austria; klaus.voit@boku.ac.at; 2 Department of Civil Engineering and Natural Hazards, Institute of Structural Engineering, University of Natural Resources and Life Sciences Vienna, Peter-Jordan-Street 82, 1190 Vienna, Austria; oliver.zeman@boku.ac.at (O.Z.); elisabeth.stierschneider@boku.ac.at (E.S.)

**Keywords:** rock, base material, post-installed anchor, design guideline, load-bearing capacity, partial safety factor

## Abstract

Unlike traditional base materials such as concrete or masonry, there are no guidelines for rock as a base material for post-installed anchors. The varying rock properties (e.g., rock type, discontinuities) and numerous installation parameters (e.g., embedment depth, anchor diameter) leave engineers with limited information on design resistances, leading to an uncertain basis for anchor applications in rock. To identify the key parameters that determine rock as a base material, an evaluation of rock characteristics was conducted, combined with in situ pull-out tests in different key geologies (granite, limestone, mica schist, dolomite, granulite) and discrete element modeling, which has been found to be suitable for investigating the load-bearing behavior of post-installed anchors in rock. Discontinuities were identified as the main factor influencing the load-bearing capacity of post-installed anchors in rock mass. Based on the in situ investigations, assessment methods for rock as a base material were proposed, along with corresponding resistance partial safety factors for design of 2.5, 2.0, and 1.7 for high, medium, and low levels of uncertainty regarding possible inhomogeneities. A limit value *R* ≥ 36, associated with rebound hammer assessments, was defined for the low degree of uncertainty, showing limitations for schistose rock. This is concluded by a design approach for determining design resistances of shallow fasteners in rock mass.

## 1. Introduction

### 1.1. Post-Installed Anchors in Different Base Materials

Post-installed anchors establish connections with underlying substrates and are designed for external load applications on the base material. The load-bearing capacity of these anchors depends largely on the embedment depth and the properties of the base material [1,2]. This means that these post-installed anchors with shallow embedment depths differ from geotechnical anchors, which are often embedded deeply. A differentiation between these two anchor types is crucial, as it influences the load transfer and installation characteristics. Post-installed (adhesive) anchors have smaller annular gaps—typically less than 1.5 times the nominal diameter—while geotechnical (injection) anchors have larger annular gaps [3]. Furthermore, post-installed anchors are typically used in concrete and masonry [4] whereas geotechnical anchors are anchored in rock and soil. In this work, post-installed anchors are investigated for the base material rock.

Anchoring on concrete, which is a rather homogeneous base material, is well studied with broadly existing literature, e.g., [5,6,7,8,9,10], and regulated by standards, e.g., EN 1992-4 [1]. In contrast, masonry exhibits significantly higher heterogeneity (brick material and type, mortar joint, etc.), leading to an increased number of potential base material compositions. While estimating the load-bearing capacity can be considered a standard procedure for concrete, masonry relies on a combination of standards, approval values, and on-site load tests (cf. TR 054 [2]; TR 053 [11]). The situation becomes even more complex considering rock mass as a base material, a naturally occurring material governed by inherent geological processes, e.g., [12], resulting in heterogeneous and frequently partially anisotropic properties. Both empirical values and normative regulations are absent due to a wide range of possible base material characteristics, depending on the type of rock (e.g., granite, limestone) and its associated properties (e.g., compressive strength, degree of weathering, joint structure, schistosity). Table 1 illustrates a characterization of the different base materials of concrete, masonry and rock mass.

### 1.2. Fastenings in Rock Mass

Fastenings in rock represent an interdisciplinary subject area at the interface of geology, material science and civil engineering, e.g., [14,15,16,17,18,19,20,21], concerning infrastructure constructions (e.g., tunnels, power plants, roads, railways, bridges) or simple fastenings for rock climbing [22,23]. The assessment of the load-bearing capacity of post-installed anchors in rock holds significant practical importance, reflected in recent scientific research [16,17,18,19,20,21]. Although it can be considered as an emerging research area, published knowledge is still limited due to complex and various parameters. However, existing literature is not able to provide practical methodologies for estimating the load-bearing capacity of post-installed anchors in rock mass through field-based approaches.

Recent research focused on identifying significant rock parameters to describe the load-bearing behavior for post-installed anchors in various geologies [16,17,18], which also applies for this work. Chemical anchorages were tested in rock blocks of different rock types in a laboratory environment to determine the embedment depth for steel failure as the most predictable failure mode [16]. Based on these results, suitable concrete models were applied focusing on embedment depths, bond stress and borehole or anchor diameters for rock application. Applicability is found in cases where mechanical rock characteristics prove to be similar to those of concrete, i.e., can be assumed to be homogeneous [17]. Investigating the load-bearing capacity in mostly isotropic limestone and granite, the flexural strength, porosity and compressive strength are identified as the main influencing rock parameters [18].

Investigating the relationship between load-bearing capacity of chemical anchors and rock mass properties, tests were conducted in in situ on rock mass, focusing on large embedment depths of 0.5 m to 2.5 m and anchor diameters of 22–32 mm [19]. The identified relevant rock mass properties were the rock quality designation index (RQD index) [24], geological strength index (GSI) [25] and unconfined rock compressive strength (UCS).

Comparable studies were conducted examining mechanical and chemical anchorages in rock with embedment depths from 1.2 to 6.0 m for underground excavation design. Here, the anchor load is a response of rock deformation, requiring prestressing to achieve the specified load-bearing behavior for rock mass stabilization [26]. Deeper anchorages activate larger rock areas, thereby minimizing the impact of small-scale inhomogeneities on load-bearing behavior. This stands in contrast to the shallow embedded anchors examined in this study, which are designed for external load application on rock with minimum embedment depths of a few centimeters.

Anchorage in base material rock naturally concerns various rock types, including soft, hard and stable rock, whereby separating surfaces have a decisive effect on otherwise possibly homogeneous substrates. Furthermore, rock masses are subject to external influences such as mechanical weathering [27], contributing to their overall high heterogeneity [28], which are difficult to reproduce in laboratory settings, making in situ testing preferable, e.g., in mines [20]. By determining failure loads in rock, significant differences from those in concrete were identified, resulting in simplified calculation methods for estimating the restraining rock capacity. Subsequently, in situ investigations on anchor groups were conducted emphasizing the observed fracture mechanics, including numerical analysis (FEM) [21]. The authors found that breakout cones in rock are 20–33% larger than in concrete.

To determine the load-bearing capacity and behavior of post-installed anchors in rock, both laboratory tests and FEM have been carried out [16,29,30,31]. Numerical modeling is considered, which, besides applying concrete models, is an effective approach to determine the load-bearing capacity of post-installed anchors in rock mass [16]. According to [29,30], FEM modeling is also suitable for detailed investigations of the interaction between anchor head geometry and crack propagation, respectively, and break-out behavior for undercut anchors, indicating larger rock cone sizes with higher surface friction on the anchor head. Three-dimensional finite element analysis was also applied on a larger scale to investigate the group effect for undercut anchors on rock cone failure, examining failure mechanics, pull-out forces and related rock strength parameters [31].

To simulate the mechanical behavior of discontinuous media, such as fractured rock masses, the UDEC (Universal Distinct Element Code—v7.0) [32] appears in the literature as suitable software, e.g., [33], but also for intact rock to investigate micro-structural mechanical behavior [34]. The UDEC also incorporates structural element modeling, e.g., cables and rock bolts, where [35] discussed limitations and required input parameters by calibrating numerical models with corresponding laboratory investigations. Fully grouted rock bolts were simulated by [36] with matching experimental results, while cables were used as structural elements by researchers simulating pull-out tests [37,38,39]. The UDEC has also been successfully used to investigate the deformation behavior and load distribution of anchors in rock under complex loading conditions such as seismic loads [40]. Therefore, the present study employs the UDEC for numerical investigations for advancing the understanding of anchor performance in rock mass.

Specifically, this study aims to develop assessment methods for classifying rock mass as a base material for shallow embedded post-installed anchors while also evaluating the applicability of these methods across various lithologies. These methods provide the basis for determining partial safety factors. The main aim of this work is to propose a design approach for estimating the tensile load-bearing capacity of post-installed anchors in rock mass.

## 2. Materials and Methods

### 2.1. General

This study presents a methodology based on both preliminary assessment of rock properties and direct measurement of pull-out loads. It is substantially based on findings from [14,15], complemented by additional testing. The experimental approach for all rock types involves fundamental geological investigations (e.g., visual assessment, rebound hammer, point load test, GSI, Rock Mass Rating [41]) to characterize relevant rock properties. Additionally, small-scale evaluations determine factors such as joint quantity, weathering and rock strength in the fastening area, while pull-out tension tests focus on determining the load-bearing capacities of post-installed anchors at shallow embedment depths of 70 mm. Finally, complementary numerical investigations were performed using the UDEC software [32] to enhance the understanding of the load-bearing behavior of post-installed anchors in rock masses and to validate the in situ experiments.

### 2.2. Rock Mass Substrate

For the investigations of post-installed anchors in rock, the properties of the rock mass substrate were analyzed, with particular attention to discontinuities within the rock mass. These discontinuities, such as joints, have proven to be critical for the performance of the anchors, regardless of the specific lithology [14,15]. Consequently, rock types from various lithologies in eastern Austria were considered, including granite, limestone, mica schist, dolomite, and granulite (Table 2). The rock types of granite, limestone, dolomite, and granulite represent rather homogeneous but jointed hard rock types (subsequently also referred to as massive rock types) and were investigated in [14,15]. It was found that, despite differences in their structures, these massive rock types can be categorized for the investigations of post-installed anchors in rock, as the key factor for such an evaluation is primarily the presence of discontinuities and their characteristics [14,15].

Therefore, to ensure broad applicability and define the limitations of a potential design procedure, additional testing was performed on mica schist. Mica schist is a highly foliated metamorphic rock, which cannot be categorized under the jointed hard rock types without further investigations.

**Figure 1:**
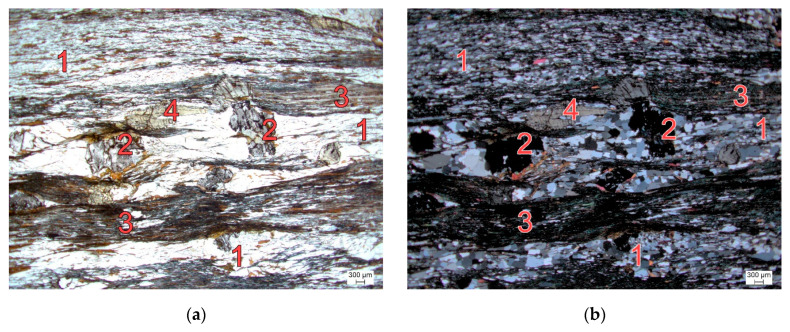
Thin section of examined rock type mica schist with (**a**) parallel nicols and (**b**) crossed nicols. 1 = quartz-rich layer, 2 = garnet, 3 = mica- and chlorite-rich layer; 4 = staurolite.

To provide further information of the structure and texture of the investigated mica schist, an examination by means of thin section analysis using a polarizing microscope (Leica DM4500P, Leica Mikrosysteme Ges.m.b.H., Vienna, Austria) was conducted. The thin section (Figure 1) shows an alternating sequence of fine-grained quartz and mica layers (composed of biotite and muscovite), each with a thickness of several hundred micrometers, forming the metamorphic schistosity of the mica schist. Between these layers, porphyroblasts of garnet and staurolite are visible, along with the presence of chlorite and plagioclase minerals.

### 2.3. Post-Installed Anchors

Fastening systems are used to connect structural or non-structural elements to a base material and are widely used in the construction industry. These are usually installed subsequently due to work processes, which is inevitably in the case of rock substrates. The connection to the base material is achieved through either mechanical interlock, friction or chemical bonding, categorizing fastening systems into mechanical and chemical anchors based on their load transfer mechanisms. The post-installed anchors investigated in this study include mechanical expansion anchors and chemical anchors, consisting of M12 threaded rods of strength class 12.9 bonded with mortar. Mechanical anchors, such as the expansion anchors studied, rely on friction to transfer loads into the base material. In contrast, chemical anchors transfer loads through bond stresses between the anchor rod and the substrate. Preliminary investigations demonstrated that, despite differences in load transfer mechanisms, the anchors—when installed with the same effective embedment depth (h_ef_) of 70 mm—can be evaluated collectively in further analyses [15].

### 2.4. Fastening Areas and Assessment Method Types

**Figure 2:**
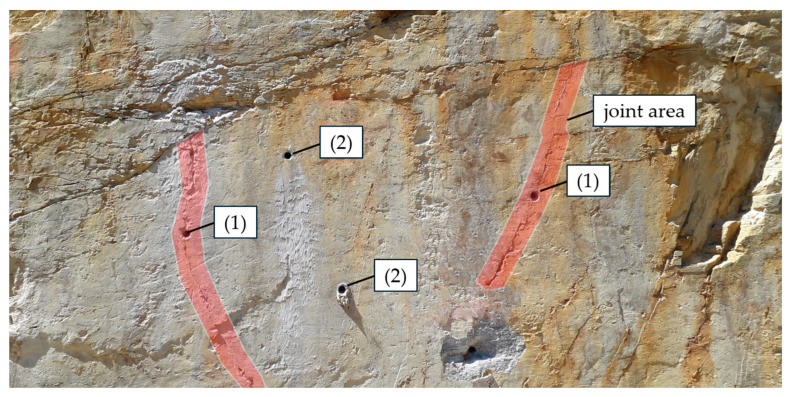
Visual base material assessment: (1) anchor influenced by visually recognizable joint (disturbed area); (2) anchor not influenced by visually recognizable joint (undisturbed area) taken and modified from [14,15].

To assess the relevant rock properties for the post-installed anchors tested, adjacent to the anchor, different assessment methods of the fastening areas were performed. These assessment methods, applied by [14,15] for jointed hard rock types, were also applied to mica schist to further advance the current understanding and limitations in formulating a guideline for assessing the load-bearing capacity of post-installed anchors in rock. In the investigations described in [14,15], a visual assessment of the fastening areas was conducted. Two distinct types of fastening areas were identified: (1) disturbed areas, characterized by visible dividing planes or joints that could influence the anchor’s performance; (2) undisturbed areas, which showed no obvious dividing planes affecting the anchor. This classification is illustrated in Figure 2 [14,15].

**Figure 3:**
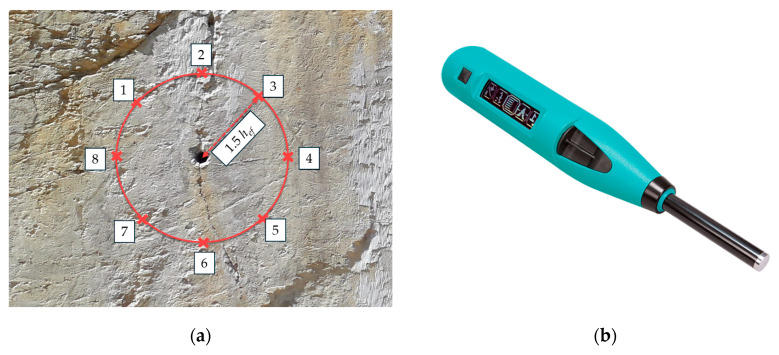
Base material assessment at the (**a**) measuring points 1 to 8 at a distance of 1.5⋅h_ef_ by (**b**) rebound hammer taken and modified from [14,15].

In the additional tests performed in mica schist, the visual assessment revealed two differing assembly situations: (1) the anchor axis parallel to foliation; (2) anchor axis perpendicular to foliation, as listed in Table 3. In addition to the visual assessment, fundamental geological investigations were performed (e.g., visual assessment, rebound hammer, point load test, GSI, Rock Mass Rating [41]). The rebound hammer assessment in the fastening area was found to be effective in determining the relevant rock properties [14]. Consequently, this study focuses on both the visual assessment and the rebound hammer evaluation. Therefore, adjacent to the anchor position, rock testing was conducted using a rebound hammer (Schmidt hammer) [51] in a circular pattern at a distance of 1.5⋅h_ef_ to the anchor (see Figure 3) [15]. The distance of 1.5⋅h_ef_ corresponds to the diameter of an idealized breakout cone of post-installed anchors in concrete for an unconfined test-setup [1].

Table 3 presents an overview of the assessments with different anchor positions and the performed number (*n*) of tests.

### 2.5. In Situ Testing of Fasteners in Different Rock Types

**Figure 4:**
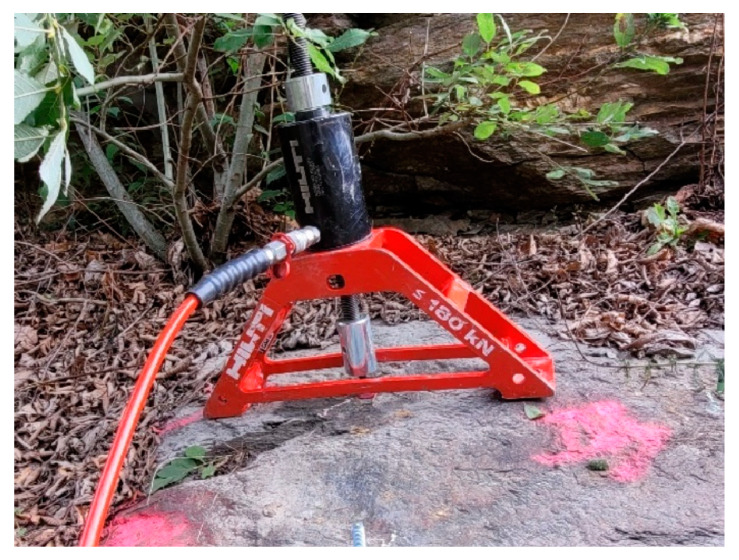
Hydraulic handpump with a wide load bridge—determination of *F_u_*.

Subsequent to the assessment of the rock parameters, pull-out tests were carried out. Figure 4 illustrates a typical installation situation of a post-installed anchor. To conduct pull-out tests on the post-installed anchors, a hydraulic hand pump featuring a wide load bridge (Figure 4) was utilized to determine the ultimate failure load (*F_u_*), without displacement recording.

### 2.6. Numerical Investigation

Complementary numerical investigations using UDEC [32] were conducted to gain a deeper understanding of the load-bearing behavior of post-installed anchors in rock mass and validate the in situ experiments, with a focus on significant discontinuities representing boundary cases. The UDEC (Universal Distinct Element Code) is a software used for numerical modeling to simulate the mechanical response of materials, including fractured rock masses. It represents the medium as an assembly of rigid or deformable blocks separated by discontinuities (e.g., joints or faults), allowing for block deformation, interaction, and movement. This method is particularly suited for analyzing geomechanical problems in highly jointed or fractured systems, e.g., [33].

In the initial modeling phase, boundary cases B1 to B8 were defined (Table 4), differing significantly in the location of the relevant discontinuity plane in the base material compared to the position of the post-installed anchor. These boundary cases were simulated numerically, but also replicated in laboratory concrete slabs to enhance model calibration and to identify application limits of field tests (rock) through laboratory experiments (concrete), as shown in [52].

The model consists of a linearly elastic isotropic block with a Young’s modulus of 57.9 GPa and a Poisson’s ratio of 0.259, including a rock bolt (chemical anchor) at the center of the block and discontinuities per boundary case according to Table 4. The behavior and material parameters of the elastic block, as well as the rock bolt (steel and mortar), were calibrated and adjusted based on experimental tests. In the initial step, steel failure was simulated to ensure the anchor failed at its calculated tensile strength, consistent with field test observations, resulting in the following material parameters: rod diameter of 10.36 mm, tensile strength of 101.16 kN and Young’s modulus of 190 GPa. Subsequently, the mortar was calibrated. To prevent pullout failure (failure of bond strength) from dominating as the primary failure mechanism and to enable various failure mechanisms of the anchor–rock–discontinuity system, the mortar was calibrated using a field test with a high pullout failure load, serving as the basis for adjusting the mortar strength. The mortar stiffness was determined using the anchor and mortar properties (anchor diameter, annular gap thickness, shear modulus) and an approximation formula [53].

The blocks were then divided along predefined separating surfaces based on boundary cases B1 to B7 (Table 4), and specific mechanical properties were assigned to the resulting contact surfaces. To prevent contact overlap and avoid program failure, the stiffness of these surfaces was set to a high value [53]. Each boundary case was analyzed as part of a parametric study, with variations applied to the discontinuity strength parameters. Initially, to assess the influence of the strength parameters, the parameter to be investigated was set to 0 while the other parameters were set to significantly high values within the same discontinuity. For instance, to assess the extent to which the tensile strength of the discontinuities impacts the load-bearing capacity and behavior of the anchorage in each boundary scenario, a tensile strength of 0 MPa was assumed. In contrast, the friction angle (50°) and cohesion (55 MPa) were assigned high values, comparable to those found in intact rock formations, to minimize their influence on the analysis [54]. Therefore, in the first step, the parameters were varied within the following ranges: tensile strength (*t*) from 0 MPa to 10 MPa, cohesion (*c*) from 0 MPa to 55 MPa, and friction angle (*φ*) from 0° to 50° [54,55,56]. These values were then adjusted for each boundary case (B1 to B7), and upon correlation, further investigated.

The edges of the block were constrained through boundary conditions applying a speed of 0 mm/s to prevent motion of the block bases and edges. Simultaneously, the rock bolt was subjected to a constant loading velocity, and a load–displacement curve was recorded. This load–displacement curve enabled the investigation of the anchor’s behavior.

In addition to the boundary cases examining significant discontinuity arrangements in the subsurface (Table 4 B1 to B7), the parametric study was extended to intact rock conditions without a dominant discontinuity (Table 4 B8). Here, a different approach was chosen for modeling. Failure of the rock mass was made possible by adding a mesh of discontinuities throughout the rock mass by Voronoi tessellation. In this approach, the intact material is divided into polygons with a predetermined size distribution and edge length, representing mineral grains that are bonded together. These grains are ideally elastic and failure occurs along the boundaries of the polygons. Here, parameters such as friction angle, cohesion, and tensile strength of the discontinuities (boundaries of the polygons) were varied for both strong and weak rock conditions. The discontinuity parameters were selected to be comparable to intact rock, as simulated in boundary case B8, using representative values from literature [25,54,57], according to Table 5. Therefore, two strength parameters (e.g., tensile and cohesive strength) were considered constant at a high (strong) and a low (weak) level, while the remaining parameter (e.g., friction angle) varied. To assess whether the joint parameters have an impact on the failure load *F_U_* in each boundary case, a percentual deviation was calculated.

## 3. Results and Discussion

The results of the in situ tests are explained below, offering insights into the load-bearing behavior and capacity through both the assessment methods and the pull-out tests. Additionally, the results from the numerical investigations are presented. Both sections demonstrate that the load-bearing capacity and behavior of the anchors are highly influenced by the presence of discontinuities and their characteristics. Subsequently, the determination of the partial safety factors and an adapted procedure for carrying out on-site tests are presented.

### 3.1. In Situ Testing of Fasteners in Different Rock Types

The investigated results of the load-bearing capacity and behavior of post-installed anchors in jointed hard rock types were presented in [14,15] and are summarized in the following section. The additional results for mica schist are also presented in the subsequent section.

In [14,15], it was found that the load-bearing behavior is primarily influenced by small-scale rock properties, typically resulting in base material failure when rebound values (*R*) indicate the presence of inhomogeneities or weak rock. Conversely, when *R* indicates fewer inhomogeneities, anchor failure mechanisms such as steel failure, pull-out, or bond failure occur [14]. Therefore, an experimental-based threshold for *R* ≥ 36, established as the 5th percentile of a log-normal distribution, was implemented enabling base material classification when combined with an engineer’s assessment [14]. It is important to highlight that the findings mentioned apply to jointed hard rock types (granite, limestone, granulite, dolomite) and remained unconfirmed for foliated rock types examining mica schist [14,15]. To assess the applicability of this conclusion to mica schist, the results for mica schist are described in more detail below, along with a comparison to granulite, which represents a jointed hard rock type.

For an assessment of the load-bearing capacity in mica schist, destructive tests were combined with geological investigations, following the approach used for massive rock. Table 6 displays the mean values and coefficient of variation (*CV*) of *F_u_* and *R* for the test series conducted on mica schist.

**Figure 5:**
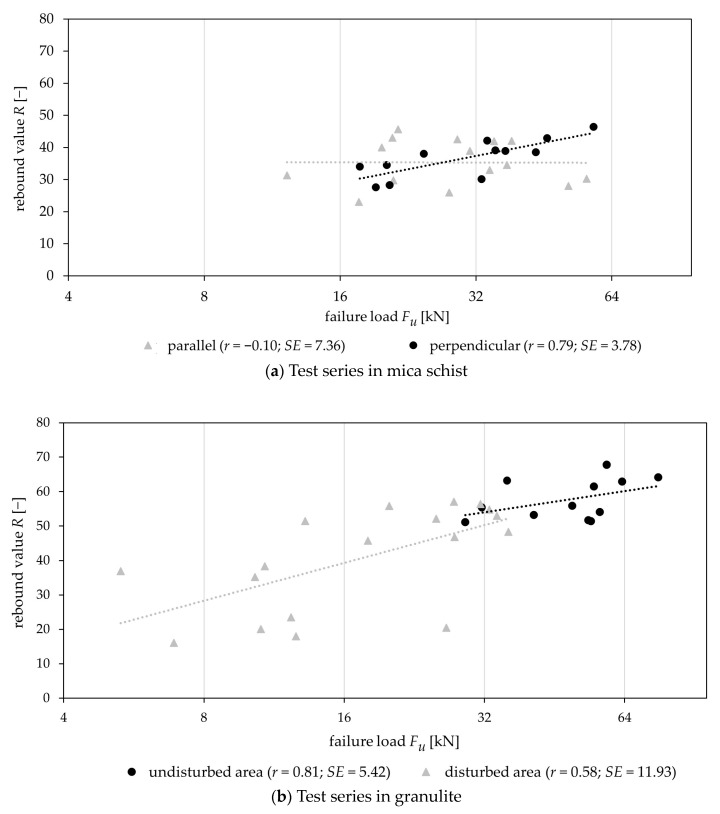
Comparing failure loads (*F_u_*) and rebound values (*R*) for (**a**) mica schist (parallel and perpendicular to foliation) and (**b**) granulite (disturbed and undisturbed) including trendlines, the correlation coefficient (*r*) and standard error (*SE*) per sample.

Figure 5 plots the relationship between the mean rebound value (Figure 3) and *F_u_* for mica schist and for granulite. For anchors with a perpendicular installation direction, a high correlation coefficient (*r*) and low standard error (*SE*) are evident (*r* = 0.79; *SE* = 3.78) compared to the parallel installation direction (*r* = −0.10; *SE* = 7.36), as shown in Figure 5a for mica schist. It can also be seen that the two samples (perpendicular and parallel installation) overlap and are not differentiable by *R* or *F_u_*. Therefore, it can be concluded that the load bearing capacity is not influenced by the installation direction and *F_u_* = f(*R*) is not valid, meaning the rebound hammer assessment reached its limitation for schistose rocks. In contrast, the test series in granulite (Figure 5b) reveal a clear distinction between samples (disturbed and undisturbed) with high *R* indicating higher load-bearing capacities and showing comparatively significant correlations (disturbed (*r* = 0.58; *SE* = 11.93), undisturbed (*r* = 0.81; *SE* = 5.42)) as shown by [14]. However, with jointed hard rock types, e.g., granulite (Figure 5b), there is also an overlapping area in which it is difficult to recognize a clear distinction between the samples. Therefore, as shown in [14], a threshold value for *R* can be used to differentiate. Furthermore, a comparison of the two rock types shows that, in granulite, the presence of discontinuities has a significant effect on load-bearing capacity. In contrast, for mica schist, the installation direction in comparison to the orientation of the discontinuities does not offer any clear indication of the possible load-bearing capacity.

These results indicate that the assessment methods used (visual and rebound hammer) are effective for jointed hard rocks like granulite but exhibit limited reliability for schistose rocks. In schistose rocks, both visual assessment of the anchoring direction and rebound values fail to provide a distinct differentiation in load-bearing capacity. In contrast, with jointed hard rock, such as granulite, a differentiation of the load-bearing capacity values is evident through visual assessment and can be differentiated even more clearly through an assessment using a rebound hammer.

**Figure 6:**
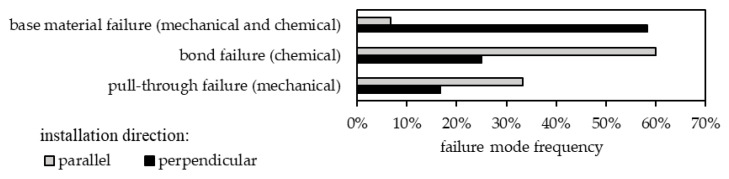
Frequency of observed failure mechanisms for perpendicular and parallel installation direction for mica schist (parallel *n* = 15; perpendicular *n* = 12).

Throughout destructive testing for mica schist, the load-bearing behavior was documented. Figure 6 indicates the occurrence frequency of failure mechanisms for anchorages parallel and perpendicular to the schistosity. Anchors installed perpendicular to the schistosity primarily typically show base material failure (58%), compared to bond failure for chemical anchors and pull-through failure (pulling the anchor through the expansion element due to insufficient expansion in the base material) for mechanical anchors. Conversely, when anchoring parallel to the schistosity, pull-through and bond failure are significantly more frequent compared to base material failure (7%). For perpendicular installation, the frequency of base material failure is similar to that observed in jointed hard rock [15], whereas for parallel installation, the frequencies differ.

**Figure 7:**
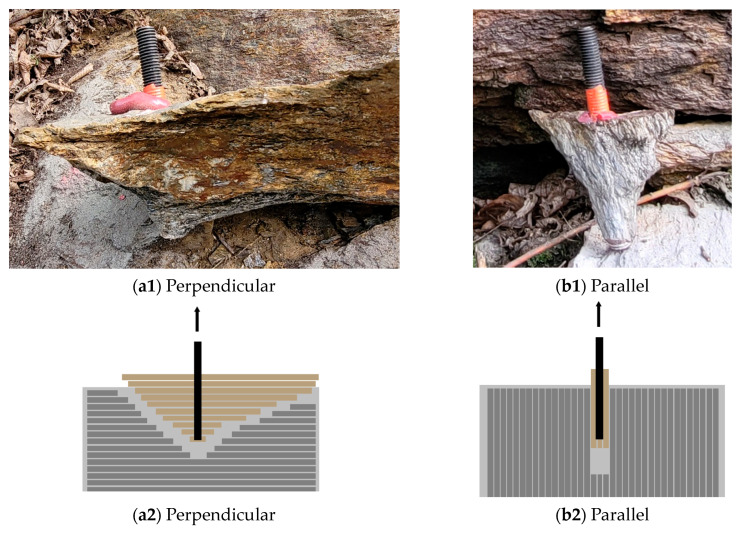
Base material failure for chemical anchors installed (**a1**,**a2**) perpendicular and (**b1**,**b2**) parallel to foliation (anchor diameter 12 mm).

While it is evident that, in mica schist, the orientation of the discontinuities does not provide any insight into the possible load-bearing capacity (Figure 5a), it does significantly affect the breakout behavior of post-installed anchors, according to Figure 7. This Figure illustrates an anchor installed perpendicular (Figure 7a1,a2) and parallel (Figure 7b1,b2) to the schistosity. When installed perpendicularly, a broad range of the base material is activated, whereas with parallel installation, only a small range of base material is engaged.

Comparing the breakout shapes of the rock types reveals that jointed hard rock types exhibit both cone-shaped excavation similar to concrete [5,15] and block-shaped volumes [15]. In contrast, perpendicular installations mainly result in cone-shaped excavation (Figure 7a1,a2), whereas parallel installations produce significantly different shapes, forming small, elongated cones along the weak planes of the schist surfaces. These variations in breakout shapes and failure mechanism frequencies indicate differences in the load transfer mechanisms for each rock type, suggesting that the load-bearing behavior is primarily influenced by discontinuities.

### 3.2. Numerical Investigation of Anchor Performance in Rock Mass

In this study, we constructed a numerical model to reflect the small-scale nature of the field experiments, where post-installed anchors with a shallow embedment depth of 70 mm and a diameter of 12 mm were tested. The investigation focused on a localized region within 1.5 times the embedment depth circular around the anchor (Figure 3a). Given this small scale, the rock in the immediate vicinity of the anchor was assumed to behave as intact rock with minimal influence from large-scale heterogeneities typically present in engineering-scale rock masses. This approach ensures that the numerical model closely replicates the conditions observed in the field tests, where the rock mass behaves similarly to intact rock due to the limited volume affected by the anchor. The influence of a single discontinuity was explicitly modeled to account for the most critical potential weakness, consistent with the single plane of weakness theory [58] and as observed in field observations.

**Figure 8:**
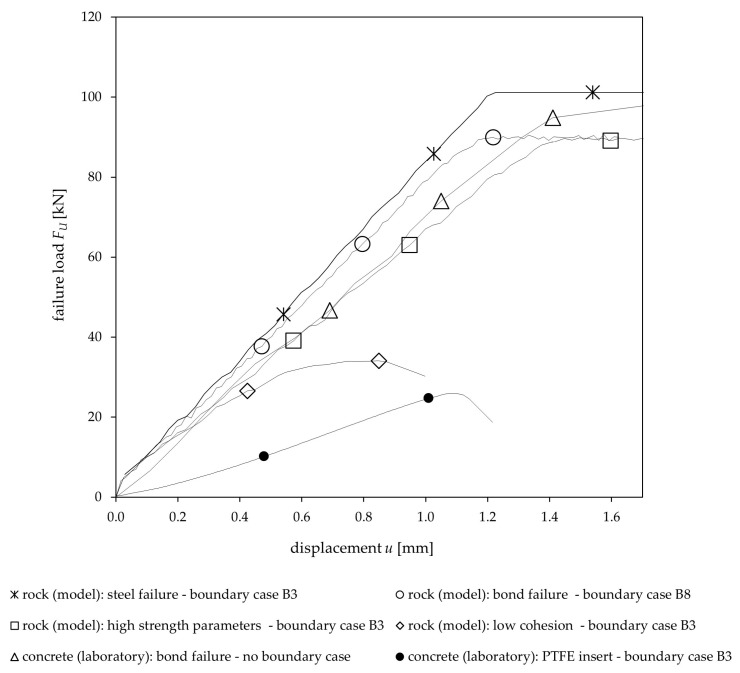
Load–displacement curves, including failure load (*F_U_*) and displacement (*u*), for various model runs representing rock and concrete tests in the laboratory.

Consequently, the load–displacement curves obtained from the numerical simulations (focusing on chemical anchors) are similar to those observed in laboratory tests (Figure 8), indicating the model’s applicability to small-scale scenarios. In engineering-scale rock masses, load–displacement behavior typically shows greater variability due to the influence of multiple discontinuities and heterogeneities, often leading to reduced stiffness and strength. In contrast, the load–displacement curves recorded during the parameter study show the higher stiffness and more predictable behavior characteristic of intact rock, also consistent with the field experiments.

These load–displacement curves were analyzed for different failure mechanisms observed in the model and compared with those from laboratory tests and are depicted schematically in Figure 8. The figure illustrates that the model’s load–displacement curves for steel and bond failure for intact rock are similar, differing primarily in the failure load (*F_U_*), while the displacement (*u*) shows only slight variation. In comparison, the load–displacement curve from laboratory tests in concrete for bond failure shows a softer yet comparable response.

In Figure 8, the load–displacement curve for rock and very high strength parameters (boundary case B3) also exhibits comparable behavior, though the anchor–rock–discontinuity system produces a softer response and shows greater displacement at a lower failure load. This suggests that not only the mortar stiffness but also the presence of a discontinuity, even with high strength properties, has a significant impact on the load-bearing behavior. In contrast, the model’s load–displacement curves for boundary case B3 with reduced discontinuity strength parameters (cohesion = 0) show significantly softer behavior. This is similar to the behavior observed in laboratory concrete tests for boundary case B3, where a low-friction insert (PTFE) was used to simulate an interface with near-zero strength, resulting in an even softer response.

Using the load–displacement curves, exemplarily shown in Figure 8, the remaining boundary cases B1 to B8 and corresponding parameter variations were analyzed. Table 7 states the percentual deviation (*F_U_*/*F_UR_*) in failure load *F_U_* when the strength values of relevant parameters are reduced, compared to the reference failure load *F_UR_*.

**Figure 9:**
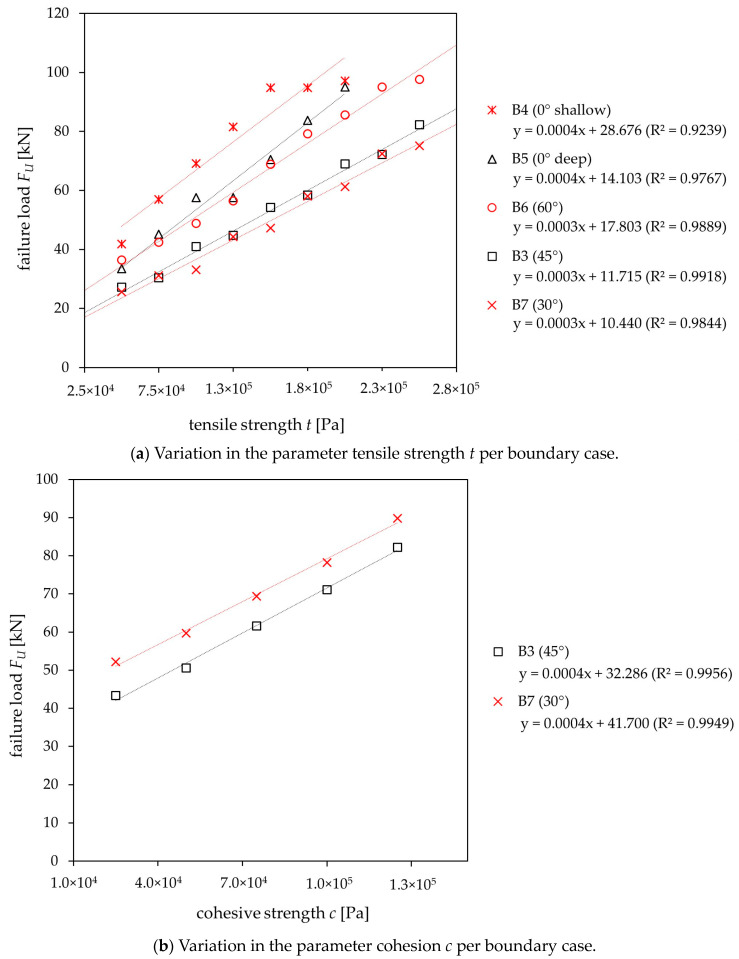
Failure load (*F_U_*) of (**a**) tensile strength *t* and (**b**) cohesion *c* for boundary cases according to Table 4 (values in parentheses refer to the angle of the discontinuities; R^2^ represents the coefficient of determination).

No substantial impact of the discontinuity on the bearing capacity was found for any of the boundary cases regarding the friction angle parameter. In contrast, an influence on the failure load was found for the tensile strength and cohesion depending on the position and orientation of the interface. For the limiting cases B3 to B7, a strong influence regarding the tensile strength was identified. Boundary cases B3 and B7 showed a significant correlation for the cohesive strength, which is why both parameters were investigated further. Figure 9a displays the parametric results for stepwise increased tensile strengths of the discontinuity and the corresponding failure loads, while Figure 9b illustrates the results for cohesion in the respective boundary cases.

Both parameters demonstrate higher failure loads with an increase in strength for the boundary cases investigated. Figure 9a shows higher failure loads for steeper discontinuity angles (30° to 60°), indicating that the tensile strength is less significant for steeper discontinuities. Otherwise, lower failure loads can be found for steeper interfaces for Figure 9b, indicating a high influence of the cohesive strength of the discontinuity for steep interfaces. Comparing boundary cases B4 and B5 in Figure 9a, whereby the discontinuities are parallel to the surface (0°), failure loads are higher for boundary case B4 (shallow discontinuity) compared to B5 (deep discontinuity).

**Figure 10:**
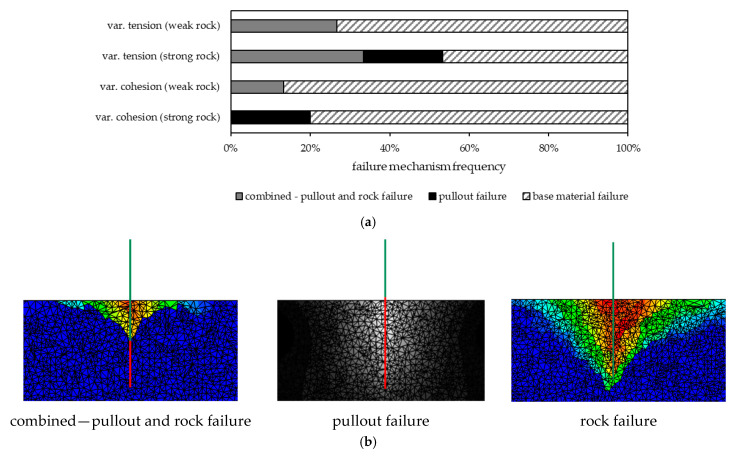
(**a**) Frequency of the failure mechanisms for different varied parameters and (**b**) illustration of the failure mechanisms for intact rock according to Table 4 B8. (**a**) Failure mechanism frequency for the variation in tensile strength and cohesive strength for strong and weak rock condition. (**b**) Illustration of the failure mechanisms for intact rock with red = high displacement to green = low displacement to blue = no displacement.

Table 7 B8 demonstrates that for intact rock, the parameters tensile strength and cohesion likewise showed a strong influence on the observed failure loads. Hence, the load-bearing behavior was investigated further for these two parameters. Figure 10 illustrates the frequency of the failure mechanisms that occurred for the different varied parameters in cases of intact rock mass.

**Figure 11:**
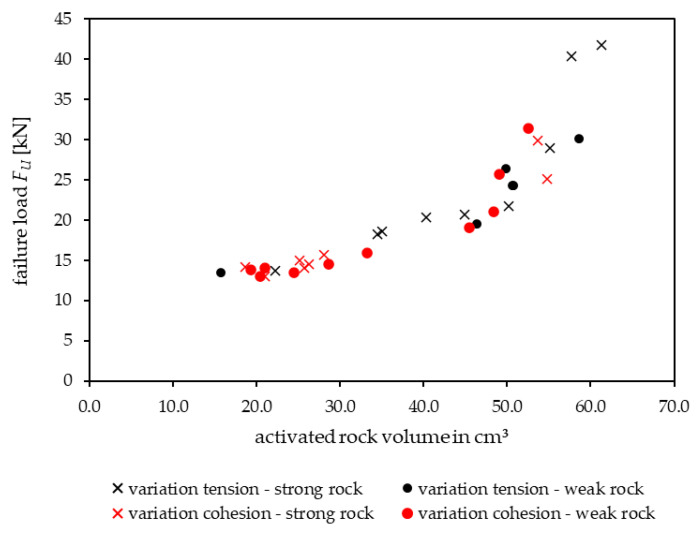
Activated rock volume and corresponding failure load *F_U_* for different variations and rock conditions.

Figure 10 illustrates that rock failure is the main failure mode for intact rock, which was also observed in the field tests for jointed hard rock types [15]. Pull-out failure appeared only under strong rock conditions, which is also consistent with the in situ experiments [15]. Figure 11 illustrates the activated rock volume, the break-out cone for intact rock (Table 4 B8) and the associated failure load. Figure 11 indicates that with higher failure loads and constant embedment depth, the activated rock volume increases regardless of the rock mass parameters.

The parametric numerical study showed that the parameters critical for failure initiation, such as load transmission, vary depending on the location and orientation of discontinuities, with the main parameters being the tensile and cohesive strength of the discontinuity. The influence for failure initiation of the tensile strength increases with shallow discontinuity angles towards the surface (30–60°), while that of cohesion decreases. These failure mechanisms observed during modeling and their frequency of occurrence are comparable with those in the field tests. Also, the load-bearing behavior and load-bearing capacity observed via laboratory and in situ-tests, have been confirmed by means of discrete modeling. Overall, modeling has demonstrated that discontinuities significantly impact the load-bearing capacity and behavior of post-installed anchors in rock mass. If the relevant discontinuities and their strength parameters—such as tensile and cohesive strength—are known, it is possible to estimate the load-bearing behavior and the failure mechanism of a post-installed anchor in the corresponding rock mass.

### 3.3. Applicability and Limitations of Assessment Methods

The observed effectiveness of visual base material assessment and rebound hammer evaluation for jointed hard rocks and the limitations observed for schistose rocks are mainly due to the differing microstructures and discontinuities in these rock types, which influence the load transfer and failure mechanisms, as also demonstrated by numerical modeling.

**Figure 12:**
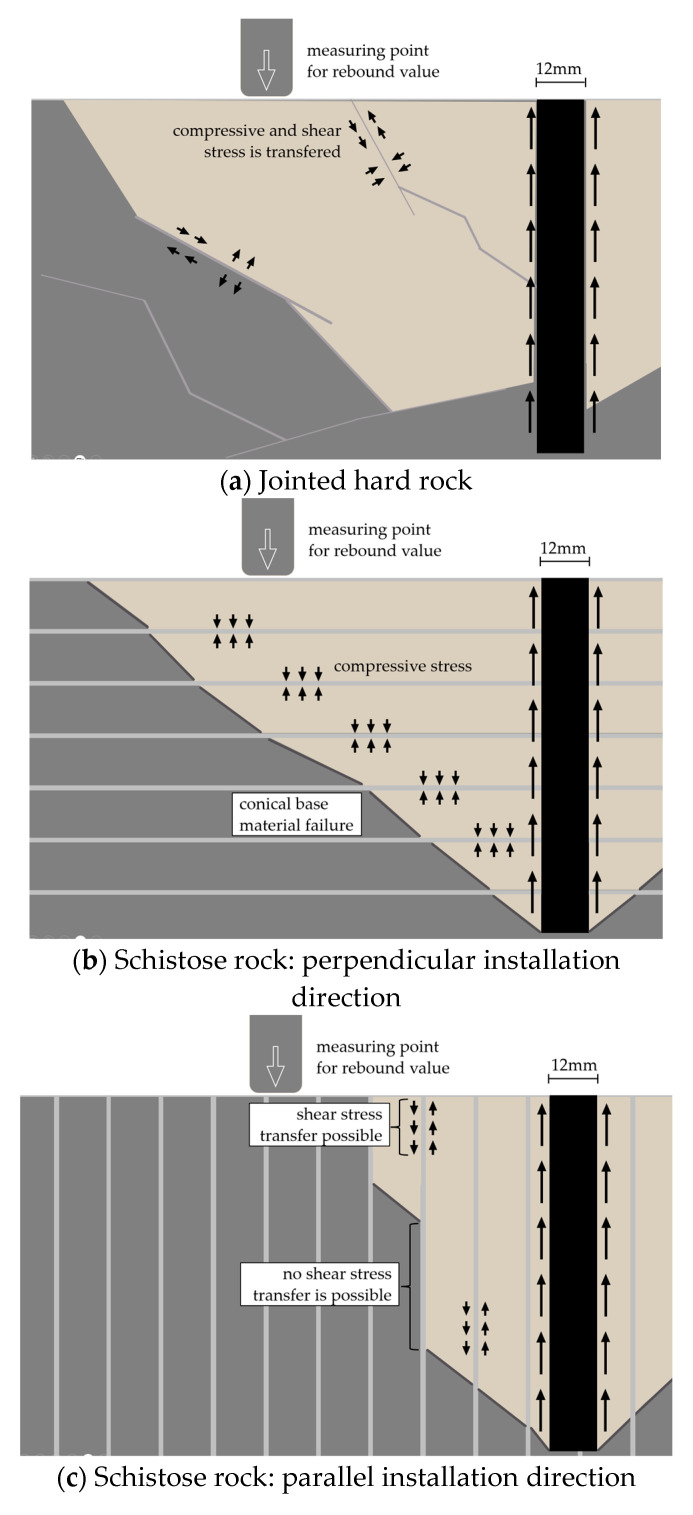
Schematic load transmission for bonded anchor type for (**a**) massive rocks and schistose rocks for (**b**) perpendicular and (**c**) parallel installation direction.

Figure 12 schematically illustrates a post-installed bonded anchor in (a) jointed hard rock and (b) schistose rock with perpendicular installation, as well as (c) schistose rock with parallel installation, displaying unaffected (gray) and affected (brown) rock areas. This illustration emphasizes how the load transfer mechanisms are influenced by the rock type and the orientation of discontinuities, which play a critical role in the surrounding rock behavior. The load bearing behavior for schistose rock suggests that in schistose rock with perpendicular installation, there is effective transfer of compressive forces between interfaces, while for parallel installation, insufficient shear stress transmission is evident. In contrast, the jointed hard rocks, when experiencing base material failure [15], exhibited conical and block-shaped breakout patterns, similar to the results observed in numerical simulations for intact rock.

In jointed hard rock types (Figure 12a), the anchor induces tensile stress into the rock, resulting in the transmission of tensile, compressive, and shear stresses between interfaces, depending on their orientation, as observed via numerical modeling. In schistose rock with perpendicular installation (Figure 12b), compressive stress and sufficient load transfer occur between interfaces, while surface rebound values provide sufficient indication of base material properties (inhomogeneities, strength). However, for parallel installation (Figure 12c), shear stress transmission between layers is insufficient in mica schist and significantly influenced by the nature of the foliated rock, with the alternating competent layers concealing surface rebound values as indicators of base material properties (e.g., different layers with different calcite contents can result in significantly different rebound values within a few millimeters), which also applies to decomposed rock.

It can therefore be concluded that existing inhomogeneities, such as discontinuities, have a significant impact on the load-bearing capacity and behavior of anchors installed in rock masses. Additionally, the methods presented for assessing rock mass as a base material, especially the assessment via a rebound hammer, are not suitable for schistose rock types, as they do not provide an indication of the relevant discontinuities.

### 3.4. Determination of Partial Safety Factors in Rock Mass

**Figure 13:**
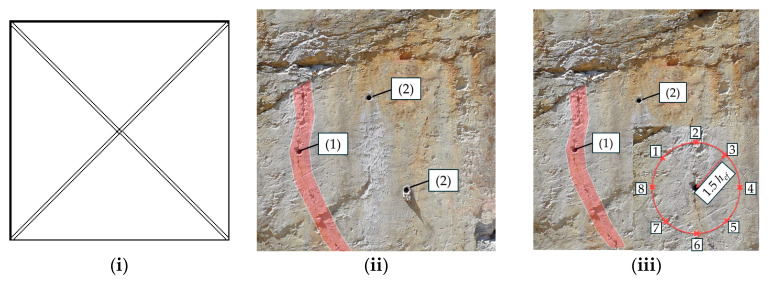
Base material assessment methods (**i**) to (**iii**) with dividing planes marked red for disturbed areas. (**i**) No examination; (**ii**) visual assessment: (1) disturbed and (2) undisturbed; (**iii**) visual and rebound hammer assessment: (1) disturbed and (2) undisturbed, 1–8 representing the rebound measurement points.

The normative regulations mentioned for designing post-installed anchors in concrete and masonry are based on a safety concept. To enable such a safety concept for post-installed anchors in rock, it is essential to define partial safety factors on the resistance side [59], therefore making the design of post-installed anchors possible. Based on the data presented, different assessment methods (Figure 13) can be distinguished depending on the level of knowledge about the rock mass as base material. Partial safety factors can be determined for these methods accordingly.

In (Figure 13i), no preliminary investigation is possible or demanded, resulting in the need to establish an increased partial safety factor *γ_R_* due to uncertainties regarding base material inhomogeneities. For the visual assessment (Figure 13ii), no visually recognizable dividing planes are supposed to influence the anchor (cf. Figure 2). To provide improved knowledge of the base material, in case (Figure 13iii), rebound values are determined according to Figure 3 combined with method (Figure 13ii). The more precise the assessment procedure is, the lower the partial safety factor on the resistance side.

Partial safety factors on the resistance side *γ_R_* are used to consider uncertainties of the involved materials and are calculated as the ratio between characteristic (*R_k_*) and design resistance (*R_d_*) [59], as shown in Equation (1). The conversion factor (*η*) is inserted to consider volume, scale, moisture and temperature effects between the construction elements and test specimens [59].

*γ_R_* = *η* × *R_k_*/*R_d_* (1)

*γ_R_*: partial safety factor [-]
*η*: conversion factor [-]
*R_k_*: characteristic resistance [kN]
*R_d_*: design resistance [kN]

Uncertainties due to the scatter of the base material properties are considered by means of distribution functions [59]. For concrete [60] and masonry [61], a logarithmic normal distribution is assumed avoiding negative strength values [62]. This is also seen as applicable to rock. The factor *η* has a value of 1.15 for concrete [59,63] and is assumed 1.00 for rock as no scale effects are considered for in situ test series in a non-laboratory environment. The characteristic resistance of material properties is calculated as the 5th percentile value using a lognormal distribution, as shown in Equation (2):

*R_k_* = *µ* × exp(−*k* × *CV*) (2)

*µ*: mean value of the lognormal distribution [kN]
*k*: percentile factor [-]
*CV*: sample coefficient of variation [%]

Table 8 presents the percentile factor (*k*) to calculate the 5th percentile based on a confidence level of 90% and an unknown standard deviation [59,64].

The factors according to Table 8 are applied and a lognormal distribution was assumed [62]. Looking at the test series per rock type, the lognormal distribution shows a goodness of fit of 50%. This does not improve with more precise methods, i.e., no improvement from method (Figure 13i) to (Figure 13iii) is observed. The factor *k* equals 1.645 for an infinite sample size as listed in Table 8.

*R_d_* is calculated based on Equation (3) as an approximation for *CV*s smaller than 20% [59]. Although the considered *CV*s in this work exceed 20%, Equation (3) is used, considering the small differences between the exact and the simplified approximation method. As a default value, the sensitivity factor *α_R_* is assumed to equal 0.8 [64,65]. If the design working life is 50 years, the reliability factor *β* equals 3.8. The design resistance *R_d_* is then calculated as follows:

*R_d_* = *µ* × exp(−*α_R_* × *β* × *CV*) (3)

*αR*: sensitivity factor [-]
*β*: reliability index [-]

By consolidating Equations (1)–(3), Equation (4) can be obtained, as shown below:

*γ_R_* = *η* × *R_k_*/*R_d_* = *η* × [*µ* × exp(−*k* × *CV*)]/[*µ* × exp(−*α_R_* × *β* × *CV*)] = *η* × exp[(−*k* + *α_R_* × *β*) × *CV*] = 1 × exp[(−1.645 + 0.8 × 3.8) × *CV*] (4)

Table 9 lists the partial safety factors for the observed massive rock types (granulite, limestone, dolomite, granite), calculated based on *CV*s corresponding to the assessment method used. The partial safety factor for method (i), where no base material assessment is carried out, is the highest at 2.41. For method (ii), which involves a visual assessment of the base material, the partial safety factor is 1.92. The lowest partial safety factor of 1.61 is observed in method (iii) where the base material is examined most extensively.

As discussed, the assessment methods (visual and rebound hammer) are applicable only to specific rock types, due to variations in rock structure. However, it should be recognized that the determination of the different partial safety factors is based on methods that gradually improve the knowledge of the base material. Consequently, the greater the anticipated uncertainties, the higher the recommended partial safety factor. Method (i) indicates high uncertainties with respect to local inhomogeneities (dividing planes). Method (ii) is less uncertain in this context, but includes an additional subjective component based on the operator’s assessment. Method (iii) involves measurement uncertainties using a rebound hammer due to weathering processes and reproducibility. Given that measurement uncertainties increase with the applied assessment methods, while the global uncertainty with regard to possible inhomogeneities decreases, the calculated partial safety factors (*γ_R_calc_*) have been rounded up to the next tenth for the proposed partial safety factors (*γ_R_prop_*) according to Table 9.

**Figure 14:**
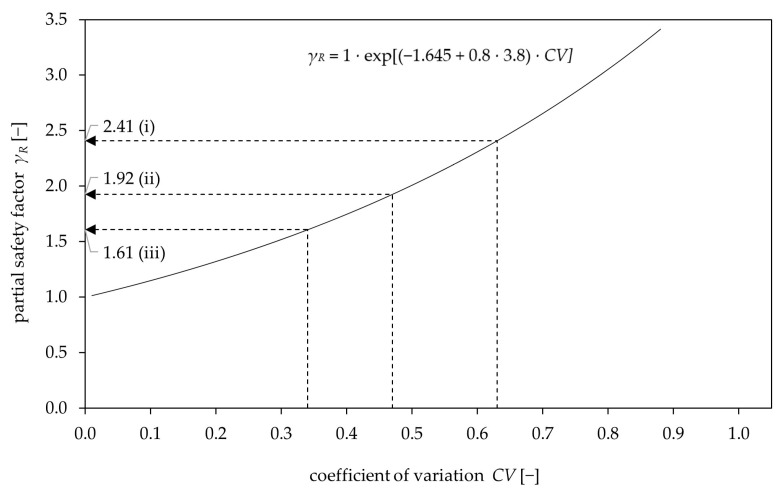
Equation (4) plotted in a diagram, indicating the relation between coefficient of variation (*CV*) and partial safety factor ≙ (*γ_R_*) for the assessment methods (i)–(iii).

Figure 14 depicts Equation (4) including the calculated partial safety factors according to the methods (i) to (iii), with partial safety factors increasing exponentially with increasing *CV* in accordance with Equation (4).

However, it is also important to address general uncertainties in measurements [65]. The Joint Committee for Guides in Metrology published a Code of Practice (COP) [66,67,68] for the determination of uncertainties for different measurement methods, which were applied to material-testing standards [69]. Studies on measurement uncertainty and its influence on “essential characteristics” of anchors in concrete have been conducted [70], which postulate that for mechanical anchors, material uncertainties are the largest contributions to the overall expanded measurement uncertainty; however, these have not been included in this paper.

### 3.5. Proposal of a Procedure for the Design of Post-Installed Anchors in Rock

Based on the determination of partial safety factors, three assessment methods have been identified, each with proposed partial safety factors. These are required to calculate characteristic and design resistances and represent the design value for engineers. To determine the characteristic resistance, “EOTA TR 053—Recommendations for job-site tests of metal injection anchors for use in masonry” [11] is applicable, which is mainly used for on-site tests in masonry. As the procedure of TR 053 is an already established assessment method, this document is considered and adapted in the following test for the base material rock. TR053 distinguishes two procedures: (A) destructive testing and (B) non-destructive testing. For procedure (A), test anchors are installed in a characteristic and representative rock section. If no representative rock sections are available (e.g., accessibility not available, limited area size), the non-destructive procedure (B) may be applied in the final installation area.

Considering procedure (A), the test anchors are loaded until failure, with a minimum sample size of *n* = 5. It is clearly pointed out that a larger sample size results in a more reliable failure load, which is therefore recommended. Other calculation methods for larger sample sizes, which are also covered by TR 053, are not considered in this proposed approach. The characteristic resistance *N_Rk1_* is determined from the mean value *N_Rm_* of the observed failure loads, according to Equation (5), as shown below:

*N_Rk1_* = *N_Rm_* × (1 − *k* × *CV*) (5)

*N_Rk1_*: characteristic resistance [kN] from procedure (A)
*N_Rm_*: mean value of the determined failure loads [kN]
*k*: percentile factor for an assumed 5% percentile, as shown in Table 8 [-]
*CV*: coefficient of variation in the failure loads [%]

To determine the characteristic resistance *N_Rk2_* for non-destructive testing (B), a proof load *N_P_* should be applied for one minute without failure, which is calculated by Equation (6), as shown below:

*N_P_* = *N_Sd_* × *γ_R_prop_* (6)

*N_P_*: proof load [kN]
*N_Sd_*: design value of stress [kN]
*γ_R_prop_*: proposed partial safety factor on the resistance side, as shown in Table 9 [-]

If no anchor failure occurred in the proof-load tests, it is possible to calculate the characteristic resistance *N_Rk2_* from Equation (7), as shown below:

*N_Rk2_* = *N_P_* (7)

*N_Rk2_*: characteristic resistance [kN] from procedure (B)

Ultimately, it is possible to calculate the design resistance *N_Rd_* according to Equation (8) with the obtained characteristic resistance (*N_Rk1_* or *N_Rk2_*), as shown below:

*N_Rd_* = *N_Rk_*/*γ_R_prop_* (8)

**Figure 15:**
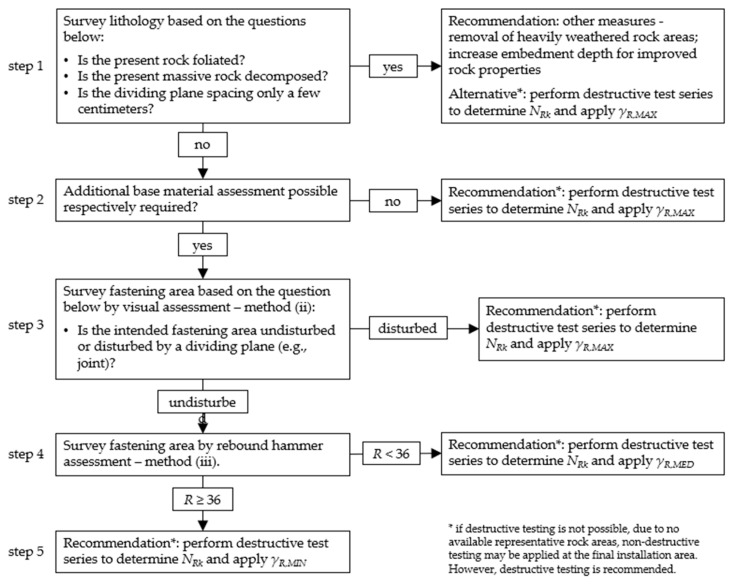
Step-by-step guideline for determining the design resistance *N_Rd_* for post-installed anchors in rock.

This procedure, adapted from TR053 [11], facilitates the determination of proof loads through on-site testing. By applying the presented assessment methods along with the corresponding partial safety factors, a step-by-step approach can be proposed to determine design resistances (Figure 15).

Initially, it is important to determine whether the rock is layered or whether the dividing plane spacing of massive rock types indicates decomposed rock, whereby *γ_R,MAX_* and a destructive test series (Figure 15 step 1) would apply. However, other measures (e.g., rock removal, increase embedment depth) are recommended due to an expected limited load-bearing capacity. In step 2 of Figure 15, if compact rock areas are observed but additional investigation is not possible, destructive test series and *γ_R,MAX_* would apply.

For non-foliated or non-decomposed rock masses, further base material assessments methods concern small-scale investigations. For visual assessment in cases of disturbed fastening areas, destructive testing and *γ_R,MAX_* would apply (Figure 15 step 3). For visually undisturbed fastening areas, further investigations by means of a rebound hammer are possible. If *R* falls below the threshold of 36 as determined in [15], destructive testing and the application of *γ*_*R*,*MED*_ are proposed. If *R* exceeds 36, *γ_R,MIN_* can be applied after a destructive test series.

In general, it can be recommended to enhance these on-site tests with numerical modeling, enabling non-testable scenarios to be simulated and thus provide more precise information about the load-bearing behavior. However, the aim of numerical modeling should not be to reduce the number of on-site tests, but rather to conduct supplementary analyses to investigate discontinuity arrangements that differ from the on-site test series.

## 4. Conclusions

This paper aims to propose a design guideline for the estimation of the load-bearing capacity of post-installed anchors in rock mass, based on engineering geological investigations and the implementation of pull-out tests in different geologies.

The results evaluated within this contribution show that the load-bearing capacity of shallow post-installed anchors is influenced primarily by rock mass properties, particularly the presence and characteristics of discontinuities. In situ experiments demonstrate a medium to high correlation (*F_u_* = f(*R*), disturbed fastening area *r* = 0.58; *SE* = 11.93 and undisturbed fastening area *r* = 0.81; *SE* = 5.42) between geological factors (discontinuities, rebound values) and the anchors’ load-bearing capacity in jointed hard rock types with wide-spaced discontinuities, but only a limited correlation in the tested schistose rock formations. In schistose rocks, the load-bearing behavior varies based on the orientation of anchor installation relative to the schistosity (perpendicular or parallel). When anchors are installed perpendicularly, base material failure is more common, occurring in 58% of cases, while anchor failure mechanisms (bond and pull-through failures) account for 42%. In contrast, for parallel installations, base material failure is rare (7%), with anchor failure mechanisms accounting for 93%. Numerical simulation via distinct element modeling supports these findings, showing that the orientation and strength of discontinuities significantly affect the load-bearing capacity and behavior. For example, tensile strength significantly influences five out of seven limiting cases, reducing the failure load by up to 83.4%, while cohesive strength affects only two cases, with a maximum reduction of 64.8% for boundary cases B1 to B7. In intact rock (boundary case B8), both parameters are critical, leading to reductions in load-bearing capacity of up to 86% (tensile strength) and 87% (cohesion). The investigations for intact rock have shown that higher failure loads in cases of base material failure correspond to the activation of larger rock volumes, and vice versa, with failure loads ranging from 13.5 kN to 41.7 kN and corresponding activated rock volumes of 15.75 cm^3^ to 61.25 cm^3^. This modeling approach can complement field investigations to enhance the understanding of the load-bearing behavior.

The study also finds that both qualitative (visual assessment) and quantitative (rebound hammer) base material assessments, for post-installed anchors in rock, are possible through preliminary investigations. The base material assessment is categorized into three levels, no assessment, visual assessment, and a combination of visual and rebound hammer assessments with a threshold of *R* ≥ 36, representing increasing knowledge of the rock properties. With each level, uncertainties about potential inhomogeneities decrease, leading to graduated partial safety factors for design of 2.5, 2.0, and 1.7, respectively. Adapting existing guidelines from masonry to rock, the study outlines two testing approaches—destructive and non-destructive—that could be conducted on-site. An approach for a step-by-step procedure is proposed, offering a framework for estimating the design resistances of post-installed anchors in rock.

As far as the content of future research is concerned, a validation of the presented design procedure for other rock types is recommended. The long-term behavior of the post-installed anchors in rock due to influences from weathering should also be the focus of future research. Finally, it should be noted that the proposed concept is based on a limited number of tests and data. Therefore, the presented concept should be considered with the character of a proposal and is intended to serve as a first guidance for engineers to determine design resistances for post-installed anchors in rock that are in accordance with the Eurocode design concept in the future.

## Figures and Tables

**Table 1:** Overview of base materials of concrete, masonry and rock mass for post-installed anchors.

	Concrete	Masonry	Rock Mass
Base material scheme:	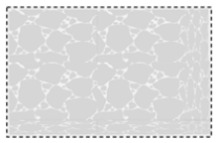	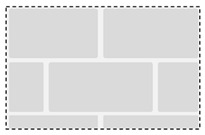	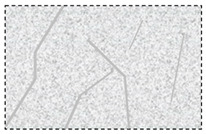
Base material characteristic:	Homogenous	Heterogeneous Systematic	Heterogeneous To a certain degree systematic
Main material parameters:	Concrete strength	Stone type including material Stone geometry	Rock type Rock strength Joint influence
Design regulations:	EN 1992-4	EOTA TR054	Not available
References:	[1,13]	[2,4,11]	[14,15,16,17,18]

**Table 2:** Investigated lithologies located in Austria.

Rock Type	Location (Austria)	Geology (Source)
Granulite	West of Vienna	Metamorphic; narrowly fractured [42]
Dolomitic limestone	East of Vienna	Sedimentary; tectonically strongly influenced [43,44]
Dolomite	Southwest of Vienna	Sedimentary; narrow joint distance [45,46]
Granite	Northwest of Vienna	Metamorphic; tectonically influenced [47,48]
Mica schist	Northwest of Vienna	Metamorphic; foliated, schistose [49,50]

**Table 3:** Test program—rebound value (*R*) and failure load (*F_u_*) assessed per anchor for different rock types and fastening areas.

Rock Type	Fastening Area (Assembly Situation)	Assessments per Anchor Position	References
Granulite	Disturbed (*n* = 18) Undisturbed (*n* = 12)	*R*, *F_u_*	[14,15] ^2^
Dolomitic limestone	Disturbed (*n* = 15) Undisturbed (*n* = 15)	*R*, *F_u_*	[14,15] ^2^
Dolomite	Disturbed (*n* = 10) Undisturbed (*n* = 0) ^1^	*R*, *F_u_*	[14,15] ^2^
Granite	Disturbed (*n* = 15) Undisturbed (*n* = 15)	*R*, *F_u_*	[14,15] ^2^
Mica schist	Parallel to foliation (*n* = 15) Perpendicular to foliation (*n* = 12)	*R*, *F_u_*	Performed for this study

^1^ No undisturbed areas could be identified; ^2^ results included from a previously published reference.

**Table 4:** Boundary cases B1 to B8 for numerical simulation with different discontinuity angles (red) and positions compared to the anchor axis (black).

Boundary Case	Schematic Illustration	Boundary Case	Schematic Illustration
B1	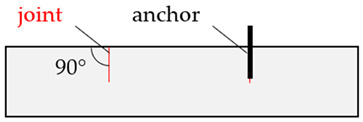	B5	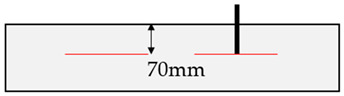
B2	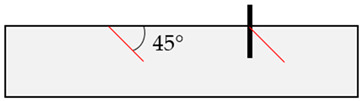	B6	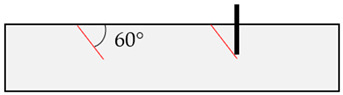
B3	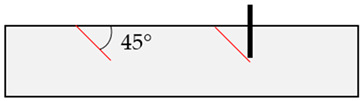	B7	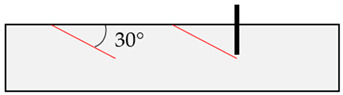
B4	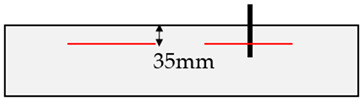	B8	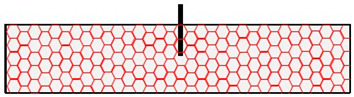

**Table 5:** Parameter variations for boundary case B8 for the parameters of tensile strength *t*, cohesive strength *c* and friction angle *φ*.

Rock Conditions	Parameter Varied	*t* in MPa	*c* in MPa	*φ* in °
	*c*	5.0	0–10	50
Strong	*t*	0–10	10.0	50
	*φ*	5.0	10.0	5–50
	*c*	0.1	0–10	30
Weak	*t*	0–10	1.0	30
	*φ*	0.1	1.0	5–50

**Table 6:** Comparison of failure loads (*F_u_*) and rebound values (*R*) per test series for mica schist.

Fastening Area	*R*		*F_u_*	
	Mean Value [-]	*CV*	Mean Value [kN]	*CV*
Parallel to foliation (*n* = 15)	35.3	20.2%	30.3	40.8%
Perpendicular to foliation (*n* = 12)	36.7	16.1%	32.4	38.9%

**Table 7:** Percentual deviation (*F_U_*/*F_UR_*) for boundary cases B1 to B8 including the parameters tensile strength *t*, cohesion *c* and friction angle *φ* according to Table 4.

Boundary Case	*F_U_*/*F_UR_* (%) for *t*, *c* and *φ*					
	*t*		*c*		*φ*	
B1	100.0		100.0		100.0	
B2	100.0		100.0		100.0	
B3	17.6		35.2		92.8	
B4	51.6		100.0		100.0	
B5	20.1		100.0		100.0	
B6	22.0		100.0		100.0	
B7	16.6		44.5		93.2	
B8	Weak	Strong	Weak	Strong	Weak	Strong
	29–100	14–100	44–100	13–100	83–100	100–100

**Table 8:** Percentile factor (*k*) for the 5th percentile as a function of the sample size (*n*) [64].

*N*	5	10	15	∞
*K*	3.400	2.570	2.330	1.645

**Table 9:** Partial safety factors for the observed rock types based on the assessment method.

Method according to Figure 13	(i) No base material assessment	(ii) Visual assessment of the fastening area with no joints affecting the anchor	(iii) Visual assessment and assessment by rebound hammer
Note:	Covers all fastening areas (disturbed, undisturbed, foliated)	Covers undisturbed fastening areas	Covers undisturbed fastening areas that meet *R* ≥ 36
	*γ*_*R*,*MAX*_ ≙ *γ*_*R*(*i*)_	*γ*_*R*,*MED*_ ≙ *γ*_*R*(*ii*)_	*γ*_*R*,*MIN*_ ≙ *γ*_*R*(*iii*)_
*CV*	63%	47%	34%
*γ_R_calc_* (calculated)	2.41	1.92	1.61
*γ_R_prop_* (proposed)	2.50	2.00	1.70

## Data Availability

The raw data supporting the conclusions of this article will be made available by the authors on request.
